# CXCL12 enhances pregnancy outcome via improvement of endometrial receptivity in mice

**DOI:** 10.1038/s41598-021-86956-y

**Published:** 2021-04-01

**Authors:** Hwa Seon Koo, Min-Ji Yoon, Seon-Hwa Hong, Jungho Ahn, Hwijae Cha, Danbi Lee, Ji-Eun Ko, Hwang Kwon, Dong Hee Choi, Kyung-Ah Lee, Jung-Jae Ko, Youn-Jung Kang

**Affiliations:** 1 CHA Fertility Center Bundang, CHA University, Seongnam-si, Gyunggi-do, South Korea; 2 Department of Biomedical Science, School of Life Science, CHA University, Seongnam-si, Gyunggi-do South Korea; 3 Department of Biochemistry, School of Medicine, CHA University, Seongnam-si, Gyunggi-do South Korea

**Keywords:** Translational research, Molecular medicine

## Abstract

Successful pregnancy inevitably depends on the implantation of a competent embryo into a receptive endometrium. Although many substances have been suggested to improve the rate of embryo implantation targeting enhancement of endometrial receptivity, currently there rarely are effective evidence-based treatments to prevent or cure this condition. Here we strongly suggest minimally-invasive intra-uterine administration of embryo-secreted chemokine CXCL12 as an effective therapeutic intervention. Chemokine CXCL12 derived from pre- and peri-implanting embryos significantly enhances the rates of embryo attachment and promoted endothelial vessel formation and sprouting in vitro. Consistently, intra-uterine CXCL12 administration in C57BL/6 mice improved endometrial receptivity showing increased integrin β3 and its ligand osteopontin, and induced endometrial angiogenesis displaying increased numbers of vessel formation near the lining of endometrial epithelial layer with higher CD31 and CD34 expression. Furthermore, intra-uterine CXCL12 application dramatically promoted the rates of embryo implantation with no morphologically retarded embryos. Thus, our present study provides a novel evidence that improved uterine endometrial receptivity and enhanced angiogenesis induced by embryo-derived chemokine CXCL12 may aid to develop a minimally-invasive therapeutic strategy for clinical treatment or supplement for the patients with repeated implantation failure with less risk.

## Introduction

Even though a remarkable improvement of assisted reproductive technology (ART) has been achieved for the last several decades, there are still a number of infertile women experiencing frequent ART failure after repeated attempts due to many unsolved problems including repeated failure of implantation. Embryo implantation is a pivotal step in reproduction involving sequential events of complex signaling networks between the embryo and endometrium^1,2^. Prior to implantation, the endometrium undergoes spatial and temporal dynamic changes, which are driven by ovarian steroid hormones to establish sufficient endometrial receptivity^2,3^. Due to the inaccessible nature and the lack of a standard tool to examine the pre- and peri-implantation phases of pregnancy in human, it has not been possible to fully estimate the proportion of implantation failure^4^. It has been reported that success rates of embryo implantation after fresh cycles of in vitro fertilization-embryo transfer (IVF-ET) process only lie at around 25–30%^5,6^. This implies that despite the extensive improvement of assisted reproductive technology, there are still meaningful proportion of refractory infertilities suffering from repeated implantation failure (RIF) or repeated pregnancy failure indicating the embryo implantation might be the rate-determining factor in infertility^7–9^. RIF is usually defined as “failure of more than three cycles of IVF-ET in which reasonably good embryos were transferred”, which occurs in 15–20% of infertile women^10^. Among the factors that attributes to RIF; unreceptive endometrium, poor embryos, and unsynchronized maternal–fetal crosstalk; defects in endometrial receptivity accounts for the half of implantation failures in women suffering from RIF after embryo transfer^11,12^. Although there have been extensive studies to identify the multi-omic signatures of endometrium of women with RIF compared to normal women^13–15^, pathophysiological features of the endometrium with poor receptivity have not been fully elucidated yet.

Angiogenesis is a process of new vessel formation from the existing vascular structure by elongation and intussusception^16^. In particular, endometrial angiogenesis plays a critical role in various female reproductive processes including folliculogenesis, cyclic endometrial regeneration, embryo implantation and placentation^17–19^. It has been reported that endometrial vasculature affects endometrial receptivity and especially determines the endometrial response to the blastocyst at the early stage of embryo implantation^20,21^. The endometrium becomes thicker with mature vascular network and increased blood flow, which reflect sufficient endometrial receptivity during the implantation^21^. Various substances including granulocyte colony-stimulating factor (G-CSF), mesenchymal stem cells (MSC), and platelet-rich plasma (PRP) have been suggested to enhance endometrial angiogenesis and receptivity for patients who are suffering from implantation failure with poor endometrial receptivity^22–24^. However, the efficacy of those substances still has not been fully evidenced yet^25–27^.

The chemokine (C-X-C motif) CXCL12, stromal cell derived factor 1α (SDF1α), and its receptors CXCR4 and CXCR7 are widely distributed in endometrial and placental tissues playing vital roles in embryonic development, implantation, placentation, and immune responses at the maternal–fetal interface^9,28,29^. CXCL12 has been reported to be expressed in human mature oocytes and fertilized egg^30^. Additionally, it promotes the migration and invasion of human first-trimester trophoblast cells inducing placental angiogenesis to form maternal decidual vasculature via its interaction with endometrial CXCR4 and CXCR7^31^. Even though several recent studies have suggested the critical roles of CXCL12 in female reproductive processes, there are few reports on the assessment of potential capacity of this chemokine CXCL12 as a therapeutic intervention to directly improve the implantation and pregnancy rates, which might be applicable for the patients suffering from repeated failure of implantation. In this study, we validated CXCL12 as a secreted factor from the embryo and explored the impact of intrauterine CXCL12 treatment on the endometrial receptivity and the induction of endometrial angiogenesis. In addition, we further examined the effect of CXCL12 on improvement of the rates of embryo implantation and pregnancy.

## Materials and methods

### Animals

Six-week-old C57BL/6 female and seven-week-old male mice (Orientbio, Gapyeong, Gyeonggi, South Korea) were used for the analyses and evaluation of impact of CXCL12. All animal breeding and experimental procedures were performed in accordance with the policies and regulations of the CHA University Institutional Animal Care and Use Committee. All reported animal experiments were approved by the CHA University Institutional Animal Care and Use Committee (IACUC, approval number 190126). All mice were housed under standard environmental conditions of 12 h light: 12 h dark at a controlled room temperature (20–22 °C and 40–60% humidity) and fed ad libitum. The study on animals was carried out in compliance with the ARRIVE guidelines Essential 10.

### Cell culture and CXCL12 treatment

Human endometrial epithelial Ishikawa cells (ATCC) and CRL4003 cells, kindly gifted from the laboratory of Professor Haeng-Seok Song of CHA University, were maintained in DMEM/F12 media (Gibco, Grand Island, NY, USA) supplemented with 10% fetal bovine serum (FBS; Gibco, Grand Island, NY, USA) and 1% penicillin–streptomycin (Gibco, Grand Island, NY, USA) as previously described^32^. For tube formation and sprouting assays, Human Umbilical Vein Cells (HUVEC, ATCC) and Human Endometrial Microvascular Endothelial Cells (HEMEC, PromoCell) were maintained in EGM-2 endometrial cell growth medium 2 (Lonza) and Endothelial Cell Growth Medium MV (PromoCell), respectively. Human recombinant CXCL12 (100 ng/ml, Peprotech, USA) was used for further analyses.

### Mouse embryo collection and co-culture

For the embryo collection, female mice were super-ovulated with intraperitoneal injection of pregnant mare serum gonadotropin (PMSG-10 IU, Daesung Microbiological, Korea) and ovulation was synchronized by human chorionic gonadotropin (hCG-5 IU, Sigma, USA) injection. Females were placed singly with males of the same strain overnight. The morning of the presence of a vaginal plug was designated day 1 of pregnancy. Pregnant mice were killed on day 1. One-cell embryos were obtained from the oviduct using a 30G dissecting needle of 1 ml syringe to tear open the ampulla of the oviduct and release. Cumulus cell around of the one-cell embryo were washed with 0.1% hyaluronidase drop at 37 °C for 5–10 min and dissociated by gentle pipetting to glass pipettes. Collected embryos were washed with M2 media (Sigma, USA) supplemented with 4 mg/ml BSA and cultured in a 20 μl drop of KSOM (Millipore, USA) covered with mineral oil at 5% CO_2_, 37 °C until the blastocyst stage. Only expanded blastocysts with clearly observable inner cell mass and trophectoderm on day 5 were included in the study. Day 5 blastocysts were transferred to confluent Ishikawa cell monolayer grown on the Matrigel-coated cover glass. Co-cultures were maintained undisturbed at 37 °C in a 5% CO_2_ for 48 h, and finally fixed in 4% paraformaldehyde in PBS for 15 min at room temperature as previously described^33^.

### Sandwich-type enzyme-linked immunosorbent assay for CXCL12 detection

Embryo-derived chemokine CXCL12 protein levels in the embryo culture media were determined by sandwich-type enzyme-linked immunosorbent assays (ELISA) according to the manufacturer's instructions (R&D, USA). Two hundred microliters of embryo culture media were collected from 15 mouse embryos and pooled. Samples were assayed in triplicate. CXCL12 levels were assayed using a validated commercial ELISA (DY008: DuoSet ELISA Ancillary Reagent Kit 2, R&D, USA). The absorbance was read at 450 nm in a 96-well microtiter plate reader.

### Assessment of vessel formation

Assessment of the rates of endothelial tube formation with or without CXCL12 (0, 100 and 200 mg/ml) administration was performed. HUVECs or HEMECs were seeded (2 × 10^4^ cells/well) onto growth factor reduced Matrigel (#354230, Corning Inc., Corning, NY, USA) pre-coated wells in 100 μl of appropriate cell growth media. Following incubation at 37 °C overnight, each well was analyzed directly under a microscope. The images (10X magnification) were subsequently analyzed using Image J.

### Assessment of endothelial sprouting

As previously reported^34^, a microengineered vascular system was designed to investigate endothelial cell responses to CXCL12 in a 3-dimensional vascularized network. To fabricate the devices, SU-8 (MicroChem) was spun onto 100 mm silicon wafers to a height of 100 μm before undergoing photolithography. The wafers were developed and dried before casting. Polydimethylsiloxane (PDMS, Sylgard 184, Dow Corning) was molded to the patterned silicon wafer using a 10:1 mass ratio of elastomer to curing agent before curing at 80 °C. Molded devices were then bonded to glass coverslip using oxygen plasma for 1 min. The microfluidic devices were incubated in an 80 °C dry oven for at least 48 h to restore hydrophobicity of PDMS. The devices were sterilized by UV irradiation before use. CRL4003 (8 × 10^6^/ml) cell were mixed with fibrinogen solution (2.5 mg/ml fibrinogen (Sigma) with 0.15U/ml aprotinin (Sigma)). Thrombin (0.5U/ml, Sigma) was added to cell mixture and immediately loaded into the channel for stromal cell. Fibrinogen solution was mixed with thrombin (0.5U/ml) was applied to the vessel channel. After allowing the gels to polymerize for 3 min at room temperature, the inlet reservoirs of the cell culture medium channels were filled with EGM-2 medium, and then aspirated to fill the hydrophobic channels. Following loading all four reservoirs, HUVECs (5 × 10^6^/ml) were introduced into the media channel. The device was then tilted 90 °C in an incubator for 40 min to attach the cell mixture to the gel-media interface. After allowing HUVECs to adhere on the fibrin gel surface, the microfluidic devices were incubated for 7 days until fully lumenized microvessels had formed. In order not to disturbed by the interstitial flow, all microfluidic experiments were conducted under static condition.

### Intra-uterine infusion of CXCL12 and fertility assessment

The female mice were anesthetized via intraperitoneal injection of tribromoethanol (avertin). A vertical incision was made to expose the uterus in the abdominal wall. CXCL12 (100 ng) was prepared in 30 μl of saline and infused using 31-gauge insulin syringe into one side of mouse uterine cavities and saline was infused into the other side of horns for the control. After 8 days of CXCL12-infusion, female mice were weakly stimulated for the ovulation with 2.5 IU of PMSG and 1.25 IU of hCG injection. The presence of a vaginal plug the following morning (day 1 of pregnancy) was used as an indicator of successful mating. For the fertility assessment depending on the intra-uterine CXCL12 treatment (100 ng/ horn), both sides of uterine horns were obtained 16 days after mating for the analyses of the rates for embryo implantation and pregnancy maintenance.

### Library preparation of CXCL12-treated uterus and sequencing for RNA-seq analysis

For control and test RNAs, the construction of library was performed using QuantSeq 3′ mRNA-Seq Library Prep Kit (Lexogen, Inc., Austria) according to the manufacturer’s instructions. In brief, each 500 ng of total RNA was prepared from CXCL12 or saline-treated (3 days or 8 days) uterine tissues and an oligo-dT primer containing an Illumina-compatible sequence at its 5′ end was hybridized to the RNA and reverse transcription was performed. After degradation of the RNA template, second strand synthesis was initiated by a random primer containing an Illumina-compatible linker sequence at its 5′ end. The double-stranded library was purified by using magnetic beads to remove all reaction components. The library was amplified to add the complete adapter sequences required for cluster generation. The finished library is purified from PCR components. High-throughput sequencing was performed as single-end 75 sequencing using NextSeq 500 (Illumina, Inc., USA). The raw and normalized data have been deposited in the Gene Expression Omnibus (GEO) data base (accession number: GSE154039).

### Data analysis

QuantSeq 3′ mRNA-Seq reads were aligned using Bowtie2^35^. Bowtie2 indices were either generated from genome assembly sequence or the representative transcript sequences for aligning to the genome and transcriptome. The alignment file was used for assembling transcripts, estimating their abundances and detecting differential expression of genes. Differentially expressed gene were determined based on counts from unique and multiple alignments using coverage in Bedtools^36^. The RC (Read Count) data were processed based on quantile normalization method using EdgeR within R using Bioconductor^37^. Gene classification for Gene Ontology (GO) and pathway analysis was performed by DAVID (http://david.abcc.ncifcrf.gov/) and Medline databases (http://www.ncbi.nlm.nih.gov/). The significance cutoffs were set for fold-change (≥ 2), *P* value (< 0.05) and FDR (0.1).

### Immunohistochemistry

Histology and immunohistochemistry were performed as described^38^ by using antibodies to Integrin β3 (Cell Signaling Technology; #13166; 1:100), and SPP1 (OPN; Enzo; ADI-905-629; 1:100) and detected with HRP-conjugated anti-mouse or rabbit secondary antibodies, and counterstained with DAPI (Sigma).

### Immunofluorescence and microscopy

Immunofluorescence staining was performed as previous described^38^. Localization studies were performed using antibody to CXCR4 (Invitrogen; PA3-305, 1:100), CXCR7 (Novus; NBP2-58162, 1:100), integrin αvβ3 (Millipore; MAB1976, 1:100), HIF1α (Cell Signaling Technology; #3434, 1:50), CD31 (Abcam; ab28364, 1:100), and CD34 (Abcam; ab8563, 1:100), and further incubated with anti-rabbit IgG fluorescence (Invitrogen) or anti-mouse IgG fluorescence (Invitrogen). Cover glasses were mounted in Vectashield mountant with DAPI (Vector Laboratories) as nuclear stain. Images were captured using oil immersion 63 × objectives Zeiss 510 microscopy (Carl Zeiss MicroImaging, Röttingen, Germany) and processed using Zen software (ZEISS), and particularly images for 3-dimensional endothelial sprouting stained with Alexa Fluor 488 conjugated monoclonal antibody against CD31 (1:200) (Invitrogen) and Hoechst 33342 (1:1000) (Thermo Fisher Scientific) were captured using a confocal microscope (Olympus FV1000, Zeiss LSM 880). Z-projection of the 3D stacks of microvascular network were obtained with ZEISS ZEN lite, and further analyzed with ImageJ (National Institutes of Health, Bethesda, MD) to obtain binary images and calculate the proportion of the fluorescent pixels within the ROI of each image, deriving the angiogenic sprout area coverage in the vessel channel.

### Quantitative RT-PCR-based analysis of mRNA expression

SYBR Green (Roche, Basel, Switzerland) assay was used to quantitate endometrial receptivity- or angiogenesis-related genes in CXCL12-treated and control samples. Total RNA extracted using TRIzol reagent (Ambion, Life Technologies Corporation, CA, USA) at 1ug was converted to cDNA using M-MLV reverse transcriptase (Promega, Madison, WI, USA), dNTP (Invitrogen, Carlsbad, CA, USA) and oligo dT primer (Labopass, Seoul, Korea). With 1/10 volume of cDNA, gene expression was quantitatively analyzed. Amplifications were run in a CFX Connect Real-Time PCR Detection System (Bio Rad, Hercules, CA, USA). A DNA melting-curve was used to confirm the presence of a single PCR product in each assay. Real-time PCR results for endometrial receptivity- or angiogenesis-related genes were normalized to β-actin mRNA expression and analyzed using the ordinary one-way ANOVA analysis with Dunnett’s multiple comparison tests. Primer sequence pairs used for these analyses are shown in Supplementary Table 1.

### Transfection of cells with short-interfering RNA

Short-interfering RNA transfection was performed as previous described^33^. Ishikawa cells were plated onto 24-well plates and cultured in DMEM-F12 supplemented with 10% FBS, 1% penicillin–streptomycin. At 70% confluence, the medium was changed to DMEM-F12 without FBS, penicillin–streptomycin and cells were incubated overnight at 37 °C. Knock-down of CXCR4, CXCR7, or in combination was performed according to manufacturer’s instruction (Dharmacon Horizon Discovery, Lafayette, USA). All siRNA reagents were pools of sequences targeting each gene (Supplementary Table 2). An siRNA which does not target any known sequence in the human genome was used for control experiments. Ishikawa cells were transfected with CXCR4, CXCR7 or non-targeting siRNA (50 nM in total concentration) using the lipofectamine 3000 transfection reagent (Thermo Fisher Scientific, Waltham, USA) for 48 h before mouse embryos were transferred onto Ishikawa cells.

### Statistical analysis

Comparison groups were analyzed with unpaired Student *t* test for parametric distributions. For multiple comparisons, the ordinary one-way ANOVA analysis with Dunnett’s multiple comparison test. For all cases, a *P* value that was < 0.05 was considered statistically significant (*P* < 0.05(*), *P* < 0.01(**), *P* < 0.001(***) and *P* < 0.0001(****)).

### Consent for publication

The content of the manuscript has been approved by all the authors.

## Results

### Embryo-derived CXCL12 induced endometrial CXCR4/CXCR7

**Figure 1:**
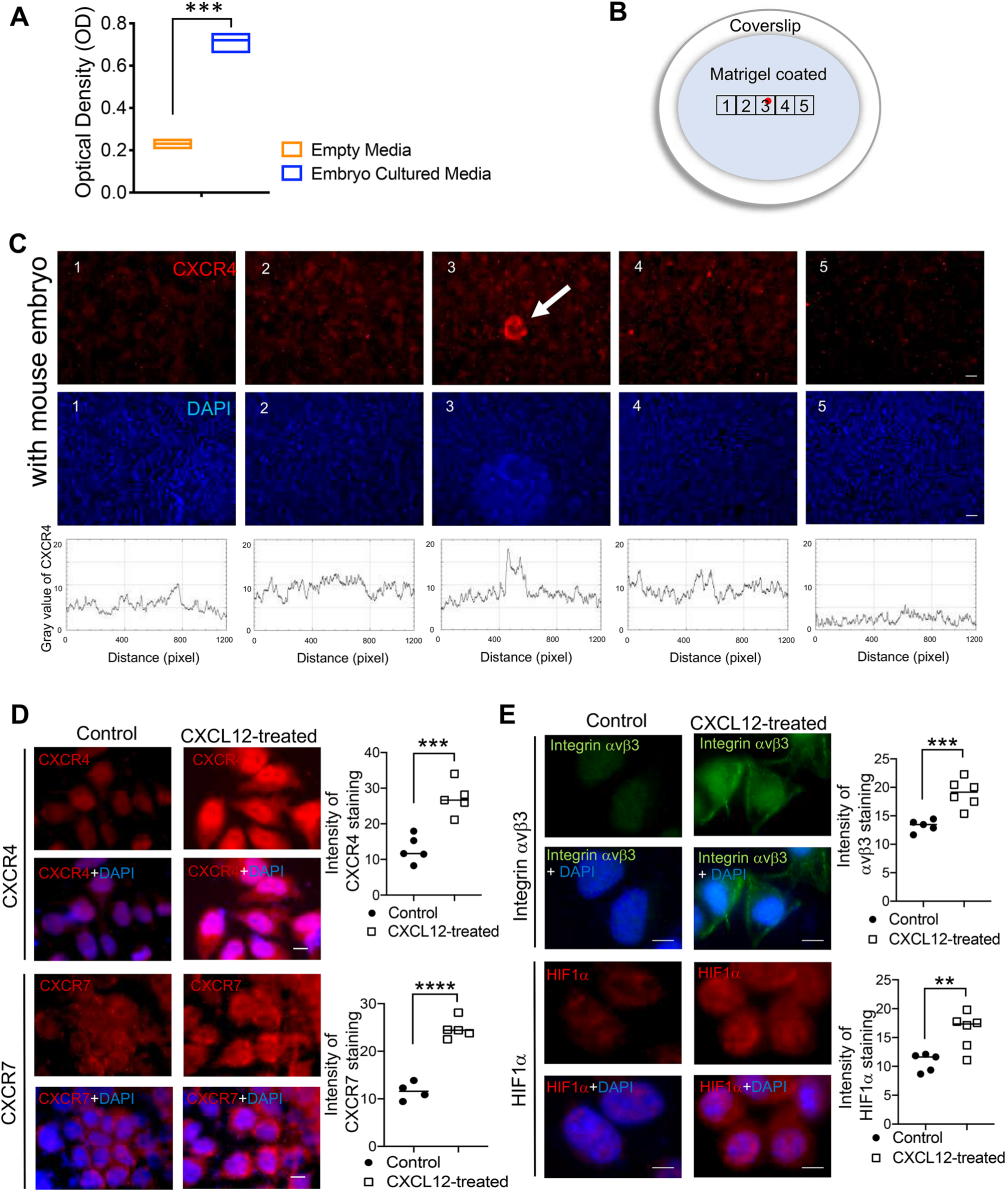
Embryo-derived chemokine CXCL12 and its receptor endometrial CXCR4/CXCR7. (**A**) CXCL12 levels were quantified in embryo culture or empty medium by ELISA. Comparison groups are from 3 independent experiments and analyzed using the unpaired Student *t* test for parametric distributions including p-values (***< 0.001). (**B**) A schematic diagram of co-culture system of mouse embryo with Ishikawa cells on Matrigel-coated coverslip. (**C**) Immunofluorescence (IF) staining images (1–5) were obtained from 5 different regions (illustrated on a diagram of (**B:1–5**)) of coverslip and mouse embryo (white arrow) was attached in the region number 3 (illustrated on a diagram of (**B**), marked as red spot). Red: CXCR4, Blue: DAPI. Scale Bar; 50 μm. Intensity of CXCR4 in each image was profiled. Images were captured at a magnification of 10 ×. IF staining of CXCR4, CXCR7 (**D**) and integrin ⍺vβ3, HIF1⍺ (**E**) in Ishikawa cells in response to CXCL12. Saline-treated cells were used for control. Scale Bar; 20 μm. Intensity of IF staining was quantified using Image J and displayed in graphs. Data are from 4 independent experiments, and analyzed using unpaired Student *t* test analysis including p-values (*< 0.05, **< 0.01, ***< 0.001, NS; not significant).

To validate the secretion of CXCL12 from the pre- or peri-implanting embryos, micro-droplets of day 3–4 mouse embryo culture media and control empty media maintained under identical culture conditions were collected and pooled up to 200 μl of volume each and CXCL12 concentration in the embryo culture media was measured using an ELISA kit (R&D, USA). Detection of CXCL12 protein in mouse embryo culture media was confirmed by the comparison of levels of CXCL12 detected in empty media (Fig. 1A). To further investigate the impact of embryo-secreted CXCL12 on the endometrial epithelium on which the implanting embryo is firstly placed inside the maternal uterus, day 5 mouse embryos were transferred to confluent Ishikawa cells and incubated for 48 h. Embryos were attached stably to the endometrial epithelial layer of Ishikawa cells. Each image including an attached embryo (red spot, white arrow) located on Matrigel-coated coverslip was shown (Fig. 1B). A well-known receptor for CXCL12, CXCR4 was locally elevated at the site of attached mouse embryo, while being unaffected further away from the attachment site (Fig. 1C). The intensity of CXCR4 expression in each image was further quantified by surface profiling (Fig. 1C). Little changes in CXCR4 expression were detected in Ishikawa cells in the absence of co-cultured embryo (Fig. S1A,B). CXCR7, a receptor of CXCL12, was also shown the increased expression near the embryo attachment site (red spot, white arrow) compared to the expression level away from the attached embryo (Fig. S1C,D). Next, CXCL12 was globally applied (100 ng/ml for 48 h) to endometrial epithelial Ishikawa cells in vitro and examined the changes in levels of CXCR4 and CXCR7, well-known receptors for CXCL12. Immunofluorescence (IF) staining analyses revealed that the expression levels of CXCR4 and CXCR7 were highly increased in Ishikawa cells with CXCL12 treatment compared to controls (Fig. 1D). Additionally, the expression levels of integrin αvβ3 and HIF1α, surrogate markers of endometrial receptivity, were dramatically increased in Ishikawa cells upon CXCL12 treatment (Fig. 1E). In particular, both expressions of integrin αvβ3 and HIF1α were elevated in the cytoplasmic compartment. These data might suggest that embryo-secreted chemokine CXCL12 increases the local crosstalk between the embryo and endometrium by activating its receptors, CXCR4/CXCR7 in the endometrium, and enhances endometrial receptivity.

### Effect of CXCL12 on endothelial angiogenesis

**Figure 2:**
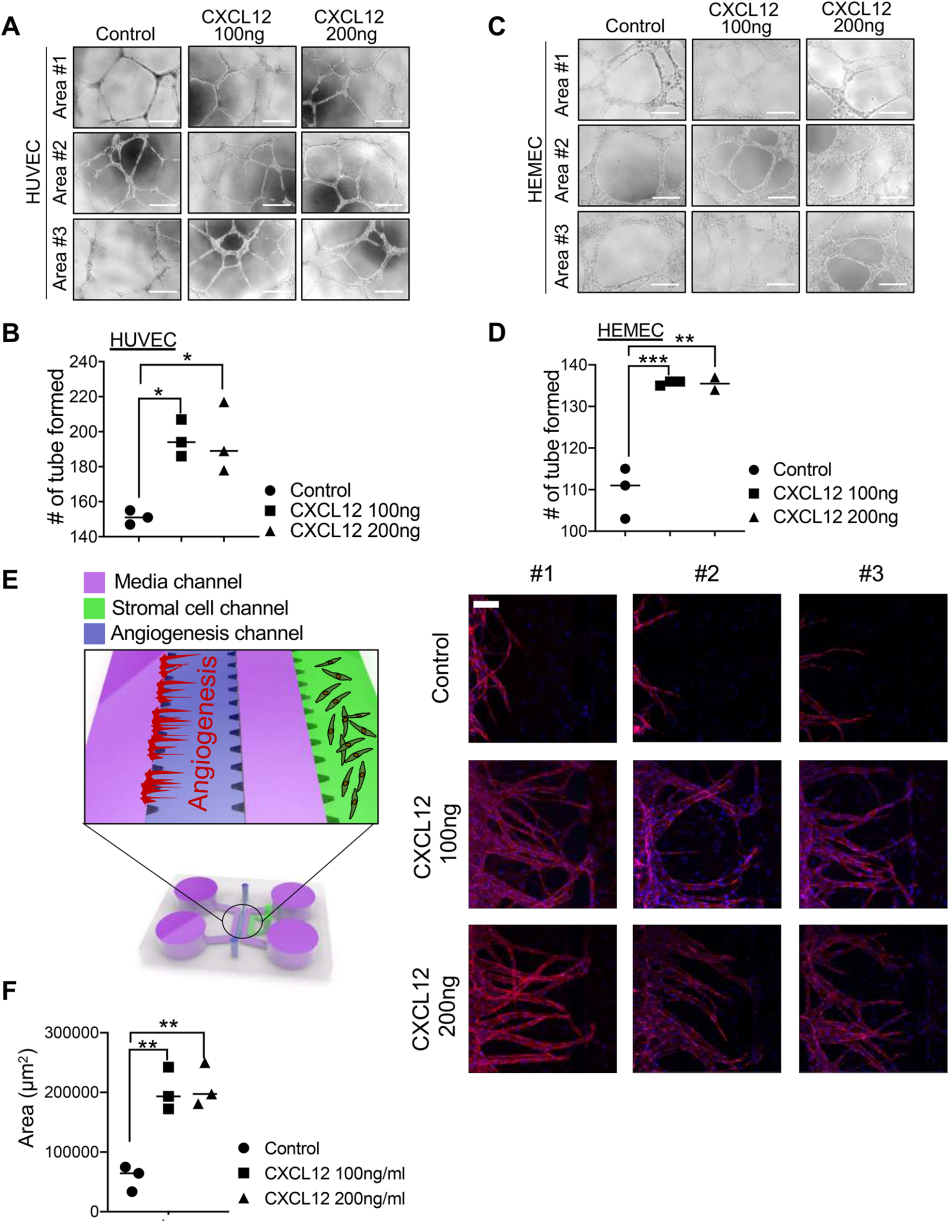
The role of CXCL12 treatment in vessel formation of vascular endothelial cells. Representative images showing tube formation of HUVEC (**A**,**B**) or HEMEC (**C**,**D**) in the absence or presence of CXCL12 (100 ng/ml or 200 ng/ml) for 48 h. Scale Bar; 200 μm. The total number of tube loops was quantified in graphs shown in (**B**) and (**D**). Data are from 3 independent experiments, and analyzed using unpaired Student *t* test analysis including *p* values (*< 0.05, **< 0.01, ***< 0.001, NS; not significant). (**E**) A micro-engineered vascular system to detect the angiogenic effect of CXCL12. Representative confocal microscopic images of angiogenic sprouting of HUVECs in response to CXCL12. Scale bar: 100 μm. The total surface area of HUVEC sprouting was quantified in a graph shown in (**F**). Data shown in (**F**) are from 3 independent experiments and analyzed using the ordinary one-way ANOVA analysis with Dunnett’s multiple comparison test including *P* values (*< 0.05, **< 0.01, ***< 0.001, ****< 0.0001, NS; not significant).

To examine the effect of CXCL12 on the ability of tube formation of human endothelial cells, we plated HUVECs (human umbilical vein cells) or HEMEC (human endometrial microvascular endothelial cells) onto Matrigel-coated plate and human recombinant CXCL12 protein (100 ng/ml or 200 ng/ml) was applied. The capacity of tube formation was quantified by measuring the total number of tube loops and branching points. These analyses revealed that CXCL12 significantly enhances the rates of tube formation of HUVECs (Fig. 2A,B) or HEMECs (Fig. 2C,D) compared to saline-treated controls. Interestingly, no significant difference was observed between the total number of tube formation at 200 ng/ml of CXCL12-treated condition and the condition of 100 ng/ml of CXCL12 treatment. Furthermore, we implemented a micro-engineered 3-dimensional angiogenesis system to explore the effect of CXCL12 on the ability of endothelial sprouting. The device consists of 5 primary channels; 2 fluidic microchannels separate 3 hydrogel-laden microchannels from each other to facilitate the supply of fresh media through the device (Fig. 2E). This multi-channel co-culture system facilitates the paracrine interaction between HUVECs and CRL4003, mediating the process of angiogenic morphogenesis between vessels and stromal layer in the endometrium. The micro-engineered angiogenesis multi-channel co-culture system was exposed to CXCL12 (100 ng/ml or 200 ng/ml). These analyses revealed that the administration of CXCL12 elevated the angiogenic effect of HUVECs displaying increased numbers and surface area of endothelial sprouting (Fig. 2E,F), which is consistent with data from tube formation assay using HUVECs or HEMECs.

### Identification of alterations in gene expression induced by intrauterine CXCL12 infusion in mice

**Figure 3:**
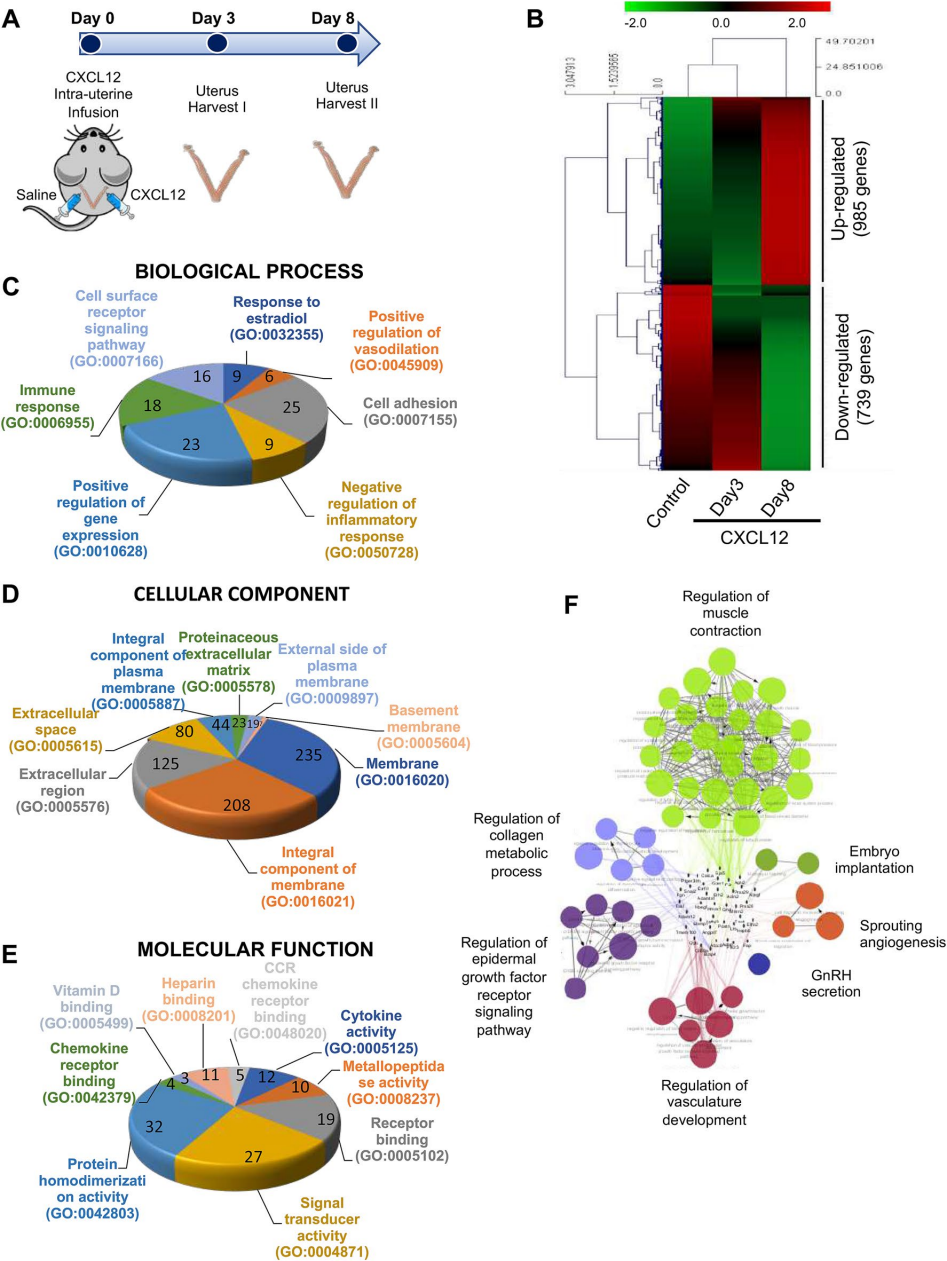
Identification of differentially expressed genes between the CXCL12-treated uterus versus control. (**A**) A schematic diagram of experimental plan of intrauterine CXCL12 administration. (**B**) Unsupervised hierarchical clustering analysis of RNA seq visualizing a heatmap plot showing differentially expressed genes (DEGs) in CXCL12-treated group vs control. Each row represents a distinct sample (6 independent CXCL12-treated groups compared to saline-treated control) and each column represents an individual gene (a list of 23,282 genes). Normalized (log2) and standardized (each sample to mean signal = 0 and standard deviation = 1) level of gene expression is denoted by color (green; low, dark; intermediate, red; high), as indicated in the gradient panel. (**C-E**) Pie charts displaying gene ontology (GO) and pathway analysis of differentially expressed genes between CXCL12-treated (Day 8) and control using DAVID tool and the numbers for gene count were indicated on each pie chart. The cutoff for significance was set by *P* < 0.05. (**C**) Biological Process (BP), (**D**) Cellular Component (CC), and (**E**) Molecular Function (MF). (**F**) A network of gene–gene interaction among CXCL12-regulated genes was constructed displaying specifically enriched signaling pathways (Cytoscape-ClueGO).

To induce the conversion of the local effect of embryo-secreted CXCL12 globally to the entire endometrium, recombinant human CXCL12 protein was infused into one side of mouse uterine horns and saline was infused into the other side of horns for the comparison (Fig. 3A). One hundred nanograms of recombinant human CXCL12 was prepared in 30 μl of saline-based solution. Leakage of intra-uterine administered CXCL12 from one side of horn to the other was prevented as possible by adjusting the appropriate volume of solution. Mice were monitored by examining dry tissue placed under the body during anaesthesia recovery^39^. Little leakage was observed. Mouse uterine tissues were obtained 3 and 8 days after CXCL12 administration (Fig. 3A). Differential gene expression data were generated from CXCL12-treated (Day 3 and Day 8) endometrial tissues compared to control (saline-treated). Unsupervised hierarchical clustering analyses using a fold change cutoff of 2 and a P-value cutoff of 0.05 identified a total of 1724 (985 genes were up-regulated and 739 genes were down-regulated) differentially expressed genes in CXCL12-Day 8 groups (Fig. 3B). To classify 1724 differentially expressed genes by their functional annotations, gene ontology (GO) and pathway analyses were performed by using the Database for Annotation, Visualization and Integrated Discovery (DAVID) online tools^40^. Enriched GO terms in each category and pathway including associated-gene counts, *P* value, and fold enrichment (FE) calculated by Fisher’s exact test and multiple comparisons test, respectively (*P* < 0.05 and FE > 1.5), are visualized in Fig. 3C,E. A total of 1724 differentially expressed genes were classified according to GO terms, including biological process, BP; cellular component, CC; molecular function, MF (Fig. 3C,E, Table 1). Whole gene ontology and pathway analyses demonstrate that CXCL12-administered uterine tissues induce the enrichment of specific BP categories, including response to estradiol (*P* = 0.00482), positive regulation of vasodilation (*P* = 0.00287), cell adhesion (*P* = 0.00203), and cell surface receptor signaling pathway (*P* = 0.000637). Moreover, enriched terms of the CC category include integral components of external side of plasma membrane (*P* = 0.00228), proteinaceous extracellular matrix (*P* = 4.47 × E^−05^), extracellular region (*P* = 9.5xE^-25^), extracellular space (*P* = 4.66 × E^−09^), and basement membrane (*P* = 0.04551). Chemokine activity (*P* = 0.0371), cytokine activity (*P* = 0.0265), signal transducer activity (*P* = 0.0222), protein homodimerization activity (*P* = 0.0195), and vitamin D binding (*P* = 0.0097) were composed of enriched MF terms. Furthermore, in-depth clustering analyses of enriched GO categories of CXCL12-treated uterus compared to saline-treated tissue were performed by ClueGO. These analyses revealed that CXCL12-induced enrichment particularly includes embryo implantation, sprouting angiogenesis, GnRH secretion, regulation of vasculature development, regulation of epidermal growth factor receptor signaling pathway, regulation of collagen metabolic process, and regulation of muscle contraction (Fig. 3F and Supplementary Table 3).

**Table 1:** Whole Gene ontology (GO) and pathway analysis of differentially expressed genes between CXCL12-day8 and control using DAVID tool. The cutoff for significance was set by *P* < 0.05, FE < 1.5. biological process (BP), cellular component (CC), and molecular function (MF).

Category	Term	Count	*P* value	Fold Enrichment	FDR
Biological process (BP)	GO:0032355 ~ response to estradiol	9	4.82E^−03^	3.421	0.08
Biological process (BP)	GO:0045909 ~ positive regulation of vasodilation	6	0.00287	6.062	0.0485
Biological process (BP)	GO:0007155 ~ cell adhesion	25	0.00203	1.979	0.0345
Biological process (BP)	GO:0050728 ~ negative regulation of inflammatory response	9	0.00191	3.971	0.0324
Biological process (BP)	GO:0010628 ~ positive regulation of gene expression	23	8.10E^−04^	2.213	0.0139
Biological process (BP)	GO:0006955 ~ immune response	18	8.05E^−04^	2.541	0.0138
Biological process (BP)	GO:0007166 ~ cell surface receptor signaling pathway	16	6.37E^−04^	2.805	0.0109
Biological process (BP)	GO:0010907 ~ positive regulation of glucose metabolic process	6	1.67E^−04^	10.97	0.0029
Biological process (BP)	GO:0071396 ~ cellular response to lipid	6	2.81E^−05^	15.36	0.0005
Cellular component (CC)	GO:0005576 ~ extracellular region	125	9.5E^−25^	2.665	1.0^−23^
Cellular component (CC)	GO:0005615 ~ extracellular space	80	4.66E^−09^	1.988	6.0E^−08^
Cellular component (CC)	GO:0005578 ~ proteinaceous extracellular matrix	23	4.47E^−05^	2.721	6.0E^−04^
Cellular component (CC)	GO:0009897 ~ external side of plasma membrane	19	0.00228	2.24	0.031
Molecular function (MF)	GO:0036094 ~ small molecule binding	9	1.30E^−06^	10.67	2.0E^−05^
Molecular function (MF)	GO:0008236 ~ serinE-type peptidase activity	14	3.84E^−04^	3.26	0.006
Molecular function (MF)	GO:0008233 ~ peptidase activity	26	0.0026	1.91	0.037
Molecular function (MF)	GO:0008201 ~ heparin binding	11	0.0067	2.763	0.094
Molecular function (MF)	GO:0048020 ~ CCR chemokine receptor binding	5	0.0059	6.773	0.083
Molecular function (MF)	GO:0030246 ~ carbohydrate binding	15	0.0031	2.484	0.044
Molecular function (MF)	GO:0005009 ~ insulin-activated receptor activity	5	2.8E−04	14.59	0.004

### Intra-uterine CXCL12 treatment increases uterine receptivity

**Figure 4:**
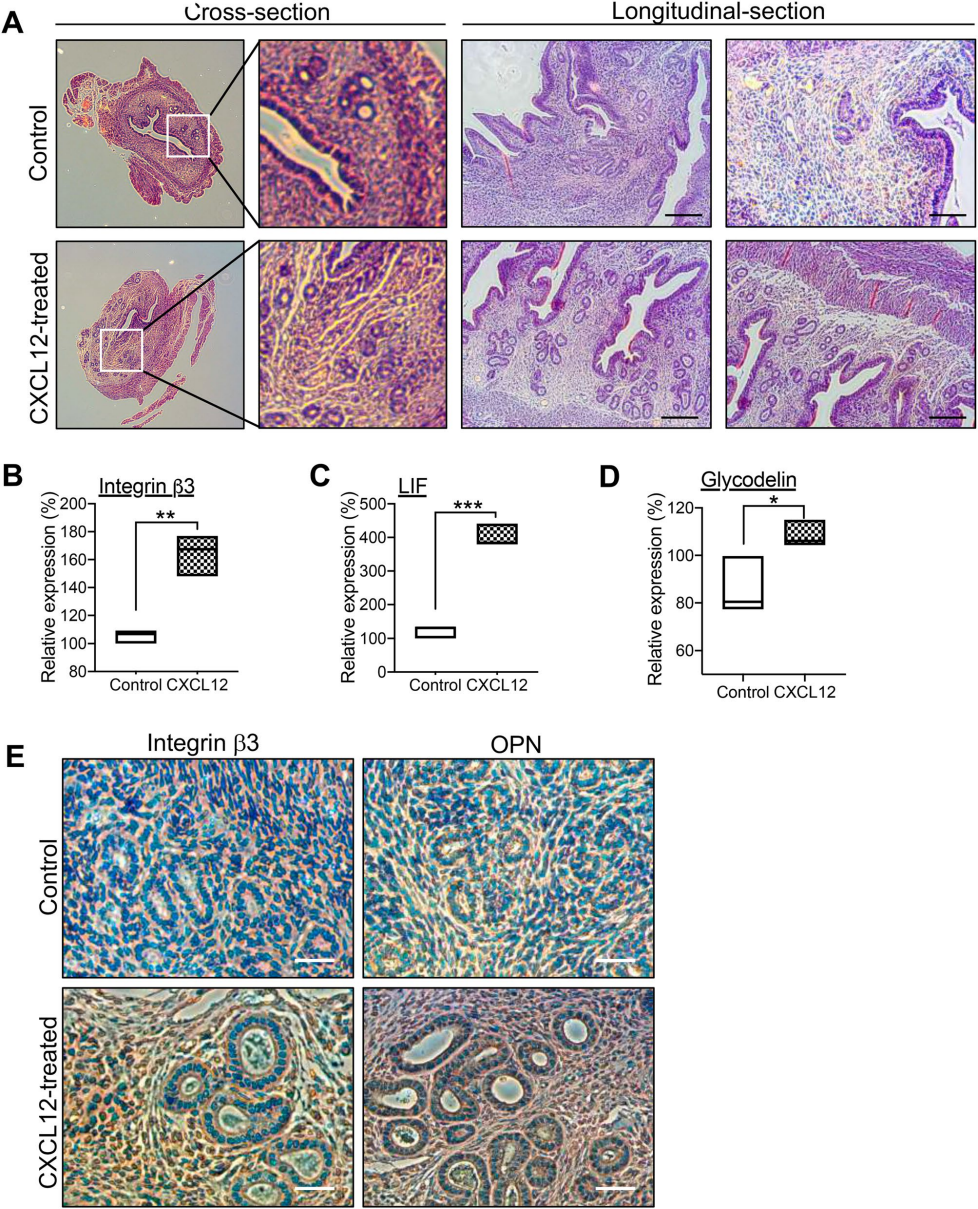
Increased endometrial receptivity with intra-uterine CXCL12 treatment. (**A**) H&E staining of cross-sectioned or longitudinally-sectioned mouse uterus harvested 8 days after CXCL12 treatment. Scale Bar; 150um. (**B-D**) QRT-PCR analysis of Itgb3, Lif, and Paep in CXCL12-treated uterus samples compared to controls. (**E**) Immunohistochemical staining of integrin β3 and SPP1 in CXCL12-treated uterus samples compared to controls. Scale Bar; 20um.

The hematoxylin and eosin (H&E) staining of paraffin-embedded uterus sections revealed that no significant histological alterations were observed with CXCL12 infusion (Fig. 4A). In order to examine the direct impacts of intra-uterine CXCL12 infusion on the alterations in global gene expression related to uterine receptivity, total RNA was extracted from the whole uterine tissues; one from CXCL12-treated and the other from saline-treated side. This comparison revealed that uterine receptivity-related markers, including *Itgb3*, *Lif*, and *Paep*^41^, showed higher expression levels in CXCL12-treated uterine tissue compared to saline-treated one (Fig. 4B,D). Our further interrogation with immunohistochemical staining of integrin β3 and one of its ligand osteopontin (SPP1) using paraffin-embedded CXCL12- or saline-treated uterine tissue sections demonstrated dramatically elevated levels of both integrin β3 and osteopontin in CXCL12-treated uterus compared to control (Fig. 4E), suggesting administered CXCL12 directly or indirectly induces integrin β3-osteopontin, adhesion molecules on the surface of endometrial epithelium in mice, which is consistent with the findings from RNA-seq analyses (Fig. 3C; BP and MF).

### Intra-uterine CXCL12 treatment promotes uterine angiogenesis

**Figure 5:**
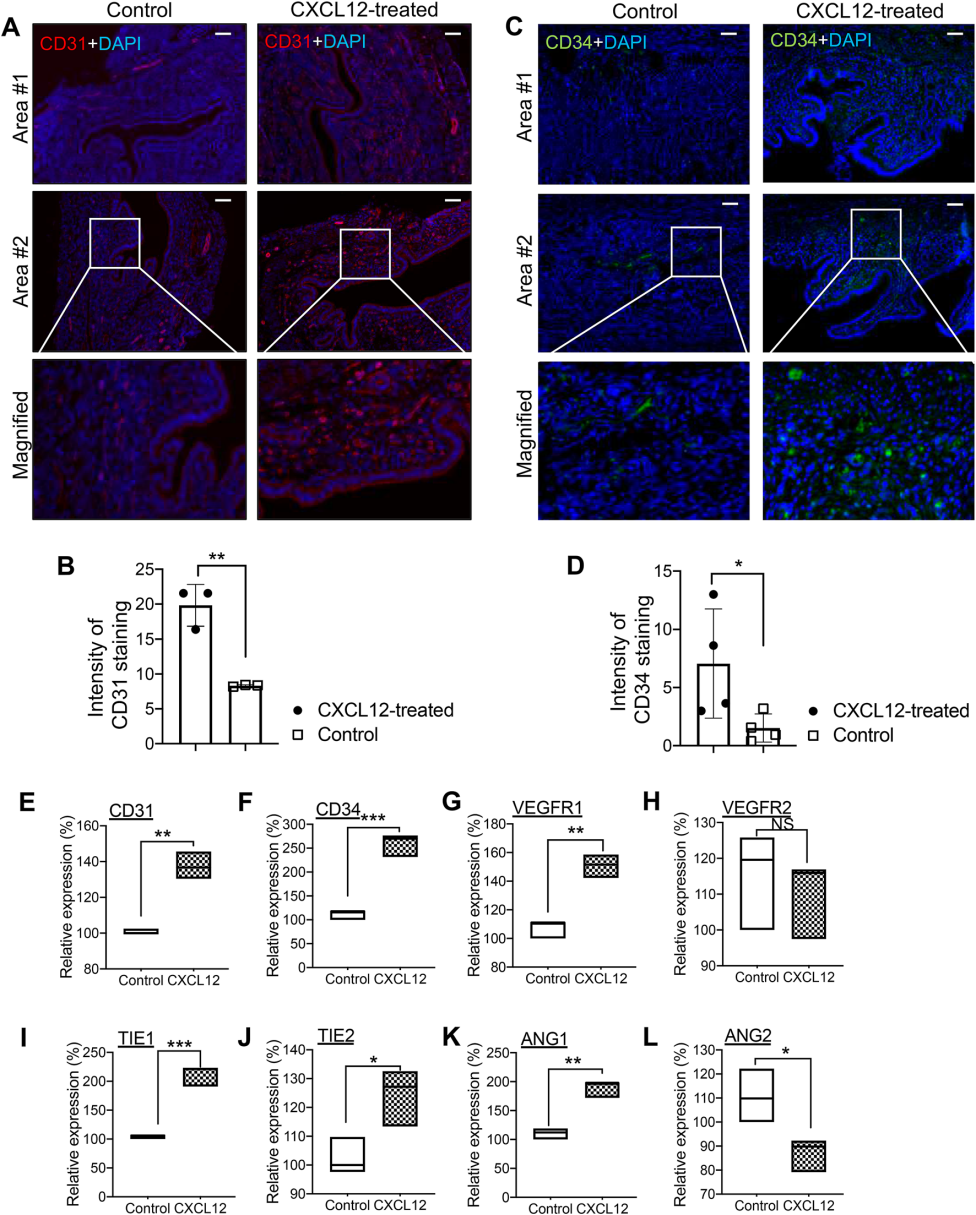
Promoted endometrial angiogenesis with intra-uterine CXCL12 treatment. IF staining of CD31 (**A**,**B**) or CD34 (**C**,**D**) in longitudinally sectioned mouse uterus harvested 8 days after CXCL12 intrauterine infusion. Saline-treated endometrium were used for control. Scale Bar; 100um. Intensity of CD31 and CD34 staining was quantified and summarized in graphs (**B**) and (**D**). Data are from 4 independent experiments, and analyzed using unpaired Student *t* test analysis including *p* values (*< 0.05, **< 0.01). (**E-L**) QRT-PCR analysis of CD31, CD34, VEGFR1, VEGFR2, TIE1, TIE2, ANG1 and ANG2 in CXCL12-treated uterus samples compared to controls. Data are from 3 independent experiments, and analyzed using unpaired Student *t* test analysis including *p* values (*< 0.05, **< 0.01, ***< 0.001, NS; not significant).

During the mid-secretory phase, the endometrium becomes mature exhibiting very coiled uterine glands with wide lumen. Increased secretion of progesterone from the corpus luteum enhances uterine thickness and blood flow to spiral arteries, which corresponds with an increased endometrial receptivity during the window of implantation^42–44^. This led us to ask whether intra-uterine infusion of CXCL12 induces endometrial angiogenesis besides the elevation of uterine receptivity by examining the expression pattern of widely-used angiogenesis markers. CD31, also known as platelet and endothelial cell adhesion molecule 1 (PECAM1), is a surrogate marker for blood vessel formation, and CD34 is a transmembrane phosphoglycoprotein and well-studied marker for hematopoietic stem and progenitor cells^44–46^. Immunofluorescence staining of CD31 (Fig. 5A,B) and CD34 (Fig. 5C,D) exhibits higher expression levels in CXCL12-treated uterus compared to control revealing CXCL12 treatment enhances new capillary formation from the pre-existing blood vessels in the endometrium. These results were validated with QRT-PCR analyses using samples obtained from the whole mouse uteruses in the presence or absence of CXCL12 intra-uterine administration. Besides of examination for CD31 and CD34, alterations in the expression levels of other well-studied angiogenesis markers including *Vegfa*, *Vegfr1*, *Tie1*, *Tie2*, *Ang1*, and *Ang2*, depending on the CXCL12 treatment were investigated (Fig. 5E,L). These revealed that CXCL12 treatment significantly induces the levels of angiogenesis-related marker molecules, except for *Vegfa* and *Ang2,* in the mouse endometrium.

### Minimally-invasive intra-uterine CXCL12 treatment improved pregnancy rates

**Figure 6:**
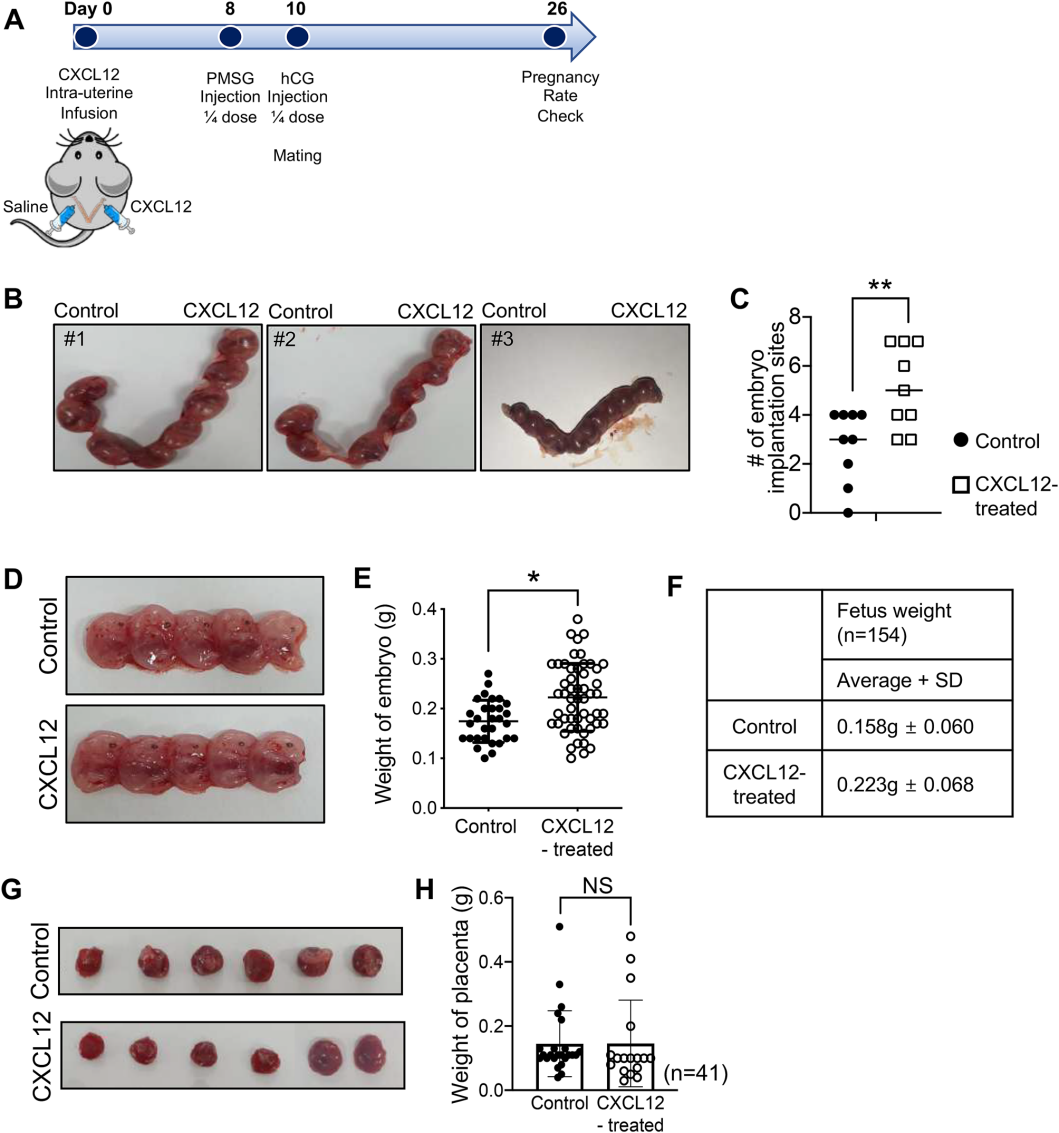
Impact of minimally-invasive intra-uterine CXCL12 treatment on the pregnancy rates. (**A**) A schematic diagram of an experimental plan for the fertility assessment followed by intra-uterine CXCL12 infusion. (**B**) Representative images of CXCL12-treated uteruses including sites of embryo implantation compared to saline-treated controls. Average numbers of embryo implantation sites were quantified in the graph shown in (**C**). (**D**) Representative images of embryos obtained from CXCL12-treated or saline-treated mouse uterus. (**E**,**F**) Average weight of embryos obtained from CXCL12-treated uterus compared to control. (**G**) Representative images of placenta derived from Control versus CXCL12-treated mouse uterus. Average weight of placenta obtained from CXCL12-treated uterus compared to control are shown in graph (**H**). Data shown for (**B-H**) are from 3 independent experiments (# of mice; 34, # of embryos; 162), and analyzed using paired *t* test analysis including p-values. (*< 0.05, **< 0.01, NS; not significant).

We next asked whether CXCL12-induced enhancement of endometrial receptivity and angiogenesis subsequently affects the pregnancy rates. CXCL12 was applied to the mouse uterus in a same manner as shown in Fig. 3A. For the fertility assessment in the presence or absence of CXCL12 treatment, after 8 days of intra-uterine infusion of CXCL12- or saline, female mice were weakly stimulated for the ovulation with PMSG (2.5 IU) and hCG (1.25 IU) injection. This assists to exclude the issues with the estrous cycle for the fertility assessment. Sixteen days after mating, both sides of uterine horns were harvested to examine the rates of embryo implantation and maintenance of pregnancy (Fig. 6A). Interestingly, the total number of implantation sites in CXCL12-treated (100 ng/horn) uterine horn was significantly higher than saline-treated side (*P* = 0.0109) (Fig. 6B,C). Little impact was observed with 10 ng or 50 ng of CXCL12 intra-uterine treatment (Fig. S2A,B). Of note, no retarded embryos were detected in both groups, however, the weight of day 16 embryos from CXCL12-treated uterus showed slightly higher (1.3-fold: 0.065 g of weight in average) than those from control group (Fig. 6D,F), while no significant differences were detected in the weight of placentas between two groups (*P* = 0.9812) (Fig. 6G,H). Moreover, particular morphological alterations at the sites of embryo implantation were rarely induced with CXCL12 treatment (Fig. S2C). Representative images of the implantation sites in mouse uteruses, embryos, and placentas obtained from different conditions are shown in Fig. 6B,D,H. Adversely, to further investigate the functional role of CXCL12 during early implantation, we applied CXCL12 neutralizing antibody (5ug/horn) into the uterine horn for 8 days and examined the rates of embryo implantation. Both sides of uterus were harvested on day 9 of pregnancy to visualize the implantation sites. We found that the number of sites of embryo implantation in control side of uterus was significantly higher than CXCL12 neutralizing antibody-treated uterine horn suggesting that suppression of CXCL12 activity with the treatment of neutralizing antibody might inhibit the embryo implantation (Fig. S2D). This led us to examine whether this CXCL12 effect is via its receptors CXCR4 and/or CXCR7. We assessed the stability of embryo attachment in vitro depending on the status of CXCR4 and/or CXCR7. A total of 44 day 5 mouse embryos were transferred to confluent Ishikawa cells, which were transfected with siRNA targeting CXCR4 or CXCR7 separately or in combination, and co-cultured for up to 48 h. Ishikawa cells transfected with siRNA targeting both CXCR4 and CXCR7 at 50 nM for 48 h showed 55 ~ 60% reduction in mRNA expression compared to control cells (Fig. S2E,F). The stability of attached mouse embryo was measured after 19 h-48 h of co-culture according to the 5-stage of standard: (1) floating; (2) weakly attached but detached after tapping; (3) weakly attached but stuck at the attachment site after tapping; (4) stably attached; and (5) stably attached and showed trophoblast outgrowth^33,47^. Embryos on control cells and cells transfected with non-targeting siControl were significantly more stably attached than embryos co-cultured with cells transfected with CXCR7 (*P* = 0.0044) and CXCR4/7 (*P* < 0.0001) siRNA in combination. However, not very significant differences were observed with CXCR4 suppression (*P* = 0.0738). This might be because of compensatory regulation among receptors for CXCL12 (Fig. S2G,H). Surprisingly, all embryos had become stably attached at 48 h in the condition of CXCL12 pre-treatment prior to siCXCR4 and/or siCXCR7 transfection even in the presence of external disturbance with tapping (Fig. S2I,J). Significantly decreased rates of embryo attachment induced by suppression of CXCR4 and/or CXCR7 were overcome with pre-treatment of CXCL12. These analyses revealed that intra-uterine CXCL12 treatment elevates the rates of embryo implantation via CXCR4/7 and maintains the pregnancy with no regression of embryos and placentas.

## Discussion

Successful implantation is a complex sequential signaling process mediated by bi-directional crosstalk between the embryo and endometrium, which is enabled by the synchronous development of the embryo and receptive endometrium especially during the early phase of embryo implantation^48,49^. Our present study identified that embryo-derived chemokine CXCL12 increases the local crosstalk between the embryo and endometrium by activating its receptors, CXCR4/CXCR7 in the endometrium enhancing endometrial receptivity and angiogenesis. This has been fully demonstrated both in vitro and in vivo by showing that the administration of recombinant CXCL12 induces cellular and molecular features of improved endometrial receptivity exhibiting remarkably elevated ITGB3, LIF, and PAEP, which are consistent with previously reported findings^50,51^. Moreover, intrauterine application of CXCL12 promoted endometrial angiogenesis, which involves the proliferation and migration of endothelial cells that are subsequently reorganized into tubular formations to form vessel networks^52,53^. This has been displayed with increased numbers of newly formed vessels accompanied by higher CD31 and CD34. Recently, it has been reported to show significantly increased CD31 expression in the mid-secretory phase of uterus in normal women compared to patients with repeated implantation failure and CD34 is expressed by vascular endothelial progenitors and endometrial stromal stem cells particularly displaying neovascular formation^26,44,46,54^. Furthermore, significantly enhanced rates of embryo implantation were observed in CXCL12-treated mouse uterine horns. Even though the detailed molecular mechanisms underlying CXCL12-induced endometrial angiogenesis and its direct or indirect correlation with improved implantation rates remain to be defined, this might suggest intrauterine application of CXCL12, which is minimally-invasive causing no traumatic injury to the endometrium at all, as a promising therapeutic strategy for patients suffering from repeated implantation failure caused by insufficient endometrial receptivity.

During the process of embryo implantation, the embryo enters the uterine cavity and adheres to the endometrial luminal epithelium inducing the local molecular and cellular alterations in the endometrium. It has been previously reported that the number of genes, including SNAI2, TGF-B1, SPARC, and Jagged1, were nominated by showing the alterations in human endometrial epithelial cells with the treatment of conditioned medium derived from human blastocysts culture following in vitro fertilization^55^. Additionally, significantly increased endometrial HOXA10 expression by blastocyst-secreted factors was observed^56,57^. Increased expression of endometrial luteinizing hormone/choriogonadotropin receptor (LHCGR) is a direct response to embryo-secreted chorionic gonadotropin and this interaction results in the expression of cyclooxygenase-2 and prostaglandin E synthase through the activation of extracellular signal-regulated protein kinase 1/2 (ERK1/2) signaling pathway^58,59^. These evidences strongly imply that embryo-derived secreted factors act as main modulators of regulating the local status of endometrial receptivity during the embryo implantation or prior to implantation. A number of chemokines, including CXCL12 and its receptor CXCR4, have been reported to be expressed at the embryo-maternal interface uterine killer cell recruitment, placentation, embryo implantation and embryogenesis^39,60^. To support these previous findings, in our current study CXCL12 has been validated as an embryo-secreted factor by detecting from the embryo culture medium and its receptor CXCR4 and CXCR7 are localized to the endometrial epithelial cells and their expressions are increased with CXCL12 treatment (Fig. 1). Dysregulation of CXCL12/CXCR4/CXCR7 axis leads to placental dysfunction by attenuating trophoblast invasion and migration, and contributes to pregnancy disorders including preeclampsia, miscarriage, and fetal growth restriction^61,62^. Although CXCL12 was detected in human first-trimester villous cytotrophoblasts, syncytiotrophoblasts, and extravillous cytotrophoblast cells, and CXCR4 expression in decidual stromal cells suggesting a crucial role of CXCL12-CXCR4 axis in the crosstalk between trophoblast cells and endometrial stromal cells during the process of embryo implantation and early pregnancy^63^, its direct clinical impact as a therapeutic intervention to improve the endometrial receptivity and pregnancy rate has not been proved yet.

Our present study showed that intra-uterine administration of CXCL12 increased endometrial receptivity and promoted endometrial angiogenesis, and it dramatically enhanced the pregnancy rates. Molecular mechanisms underlying these potential consequences might be supported by the fact that the CXCL12-CXCR4 interaction is known to promote cell–cell adhesion between the embryo and endometrium by activation of endometrial integrin family, notably alpha3, alpha5, beta1, and beta3 subunits, accompanied by increased phosphorylation of ERK1/2, JNK, and p38 pathway. This can be evidenced by our RNA-seq analyses implicating that intrauterine administration of chemokine CXCL12 might induce the intra- and inter-cellular signaling via binding to chemokine receptor CXCR4 or CXCR7 and activating cell surface receptors, such as G-protein alpha subunit, vitamin D, heparin binding, and insulin-activated receptor as second messengers to possibly result in promoting angiogenesis and mediating embryo implantation^64,65^, which is supported by enrichment of signal transducer activity classified in BP and MF categories. This eventually results in enhancement of cell–cell adhesion between the embryo and endometrium, trophoblast invasion, and endothelial angiogenesis^66,67^. Recently, it has been reported that invasive CXCL12 injection to the uterine muscle layer demonstrated a beneficial effect for endometrial regeneration on the improvement of endometrial receptivity of thin endometrium with the combined treatment with bone marrow-derived mesenchymal stem cells^68^. CXCL12 was suggested as a potent chemoattractant of bone marrow-derived mesenchymal stem cells mediating immunomodulatory actions and promoting angiogenesis^69^. Moreover, as an effective chemotactic factor for T cell, CXCL12 has been demonstrated to modulate the regulatory T cells to migrate into the pregnant uterus by binding its receptor CXCR4 and establish a beneficial microenvironment for the fetus^70^. To support these previous reports, our data showed that angiogenesis-related gene expression was significantly increased by CXCL12 and higher numbers of newly formed blood vessels were observed in CXCL12-administered mouse uterus. Consistent with previous report, CXCL12 enhanced the formation and maturation of blood vessels within the co-culture system of endometrial stromal cells with endothelial cells (Fig. 4). Of note, in our current study we applied CXCL12 into the uterine cavity with a minimally-invasive method, which is completely distinguishable to other studies, rendering dramatic impacts on improvement of pregnancy rates particularly during the embryo implantation without any retarded embryos.

## Conclusions

In summary, our present study provides a novel evidence suggesting CXCL12 treatment as an efficient therapeutic intervention to improve the endometrial microenvironment by increasing endometrial receptivity and angiogenesis. Further study will be required to discover the molecular and cellular regulation of CXCL12 to the induction of endometrial receptivity and angiogenesis, and evaluate the reproductive toxicity of intra-uterine application of CXCL12 prior to clinical trials. Even though there must be numerous factors apart from endometrial receptivity and angiogenesis to determine the success or failure of embryo implantation and pregnancy, a better understanding of endometrial receptivity and angiogenesis induced by embryo-derived chemokine CXCL12 may aid to develop a minimally-invasive therapeutic strategy for clinical supplement for the patients with repeated implantation failure with less risk.

## Supplementary Information

Supplementary Information 1.

Supplementary Information 2.

## Data Availability

RNA-seq data that support the findings of this study have been deposited in GEO with the primary accession code GSE154039. The authors declare that all other data supporting the findings of this study are available within the article and its Supplementary information files. Please contact the corresponding author for data on reasonable request.
