# Supplementary material for: CXCL12 enhances pregnancy outcome via improvement of endometrial receptivity in mice
Source: Sci Rep. 2021 Apr 1;11:7397. doi: 10.1038/s41598-021-86956-y (PMC8016928; doi:10.1038/s41598-021-86956-y)
Supplement: Supplementary file 1 — Supplementary Information 1. [file 41598_2021_86956_MOESM1_ESM.pdf]

**Embryo-derived chemokine CXCL12 enhances pregnancy outcome via improvement of endometrial receptivity in mice**

[1]Hwa Seon Koo, [2]Min-Ji Yoon, [1]Seon-Hwa Hong, [2]Jungho Ahn, [2]Hwijae Cha, [2]Danbi Lee, [1]Hwang Kwon, [1]Dong Hee Choi, [2]Kyung-Ah Lee, [2]Jung-Jae Ko, [2]Youn-Jung Kang

**Affiliation**

[1]CHA Fertility Center Bundang, [2]CHA University, Seongnam-si, Gyunggi-do, South Korea

Author for correspondence (yjkang@cha.ac.kr)

# Supplementary Figure 1

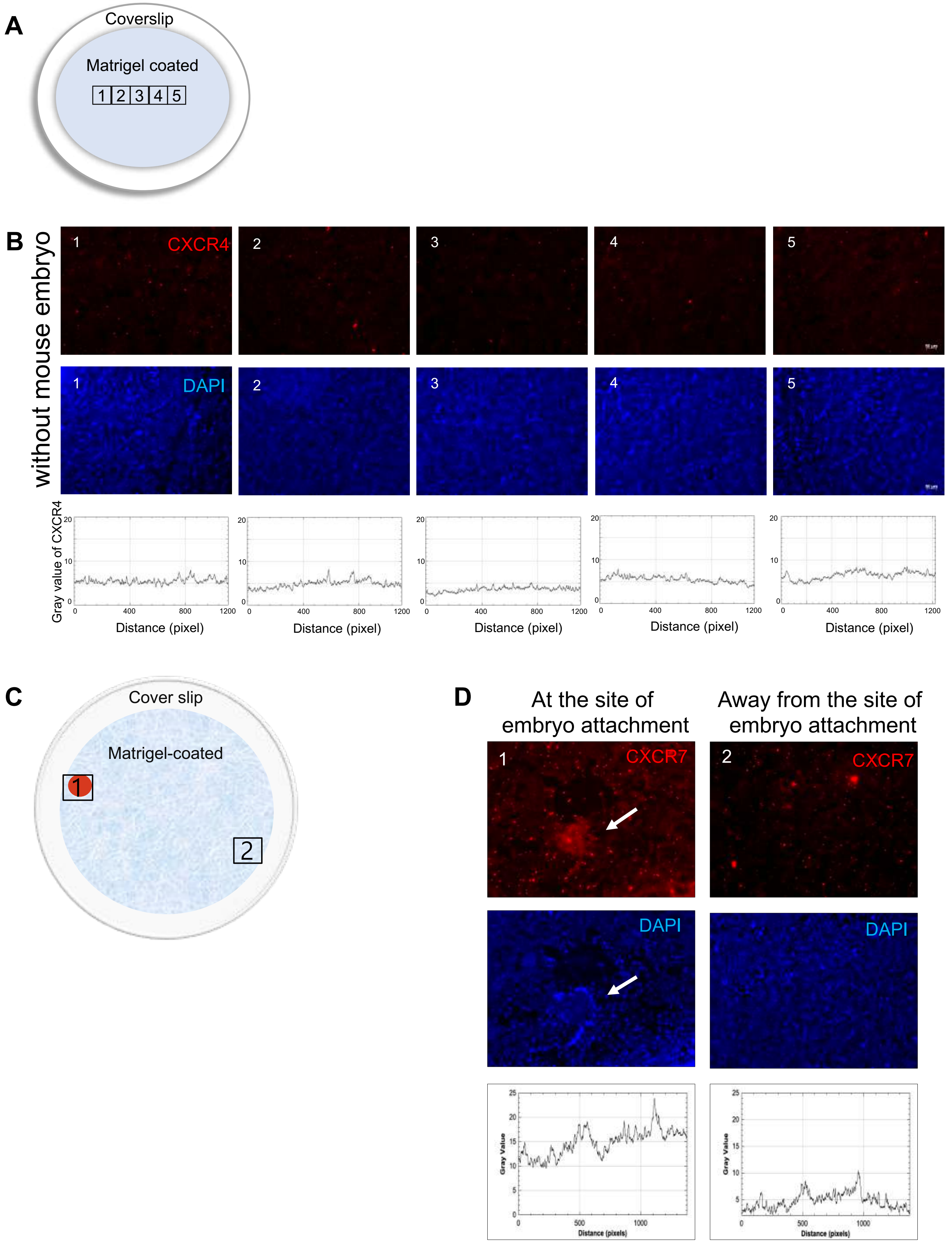

## Supplementary Figure 2

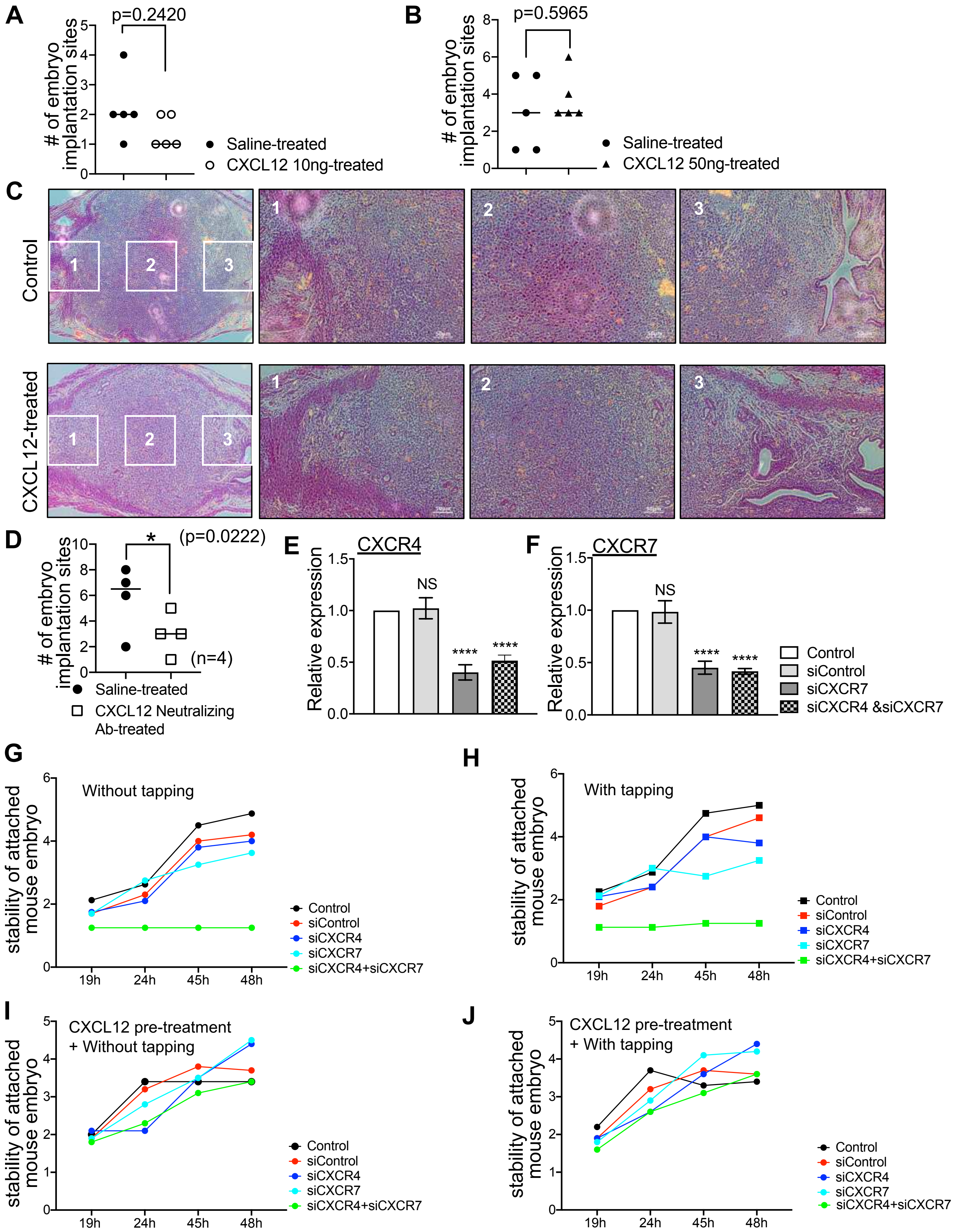

| | | |
|---|---|---|
| ITGB3 | Forward | GCTCACCTTGTCCAGAAAC |
| | Reverse | CTGAGGAAAGGGAATTGTATG |
| LIF | Forward | ATTGTGCCCTTACTGCTGCTG |
| | Reverse | GCCAGTTGATTCTTGATCTGGT |
| Glycodelin | Forward | ACTTCGCCATCATGGACTTGG |
| | Reverse | CTGGTGCGATGCCTCTTCTG |
| CD31 | Forward | GGTGCATGGCGTATCCAAG |
| | Reverse | TGGAGGTCTTATCTATCCTTCGC |
| CD34 | Forward | CTTCCCCAACTGGCATACTGC |
| | Reverse | TCCAGAGCATTTGATTTCTCCC |
| VEGFR1 | Forward | CACTGACATACCCAAACTTGTGC |
| | Reverse | GTCCCATGTTATTCTTTGCCCAT |
| VEGFR2 | Forward | CAAACCTCAATGTGTCTCTTTGC |
| | Reverse | AGAGTAAAGCCTATCTCGCTGT |
| TIE1 | Forward | GGTCACACACACGGTGAACAA |
| | Reverse | TGCCAGTCTAGGGTATTGAAGTA |
| TIE2 | Forward | CTGGAGGTTACTCAAGATGTGAC |
| | Reverse | TCCGTATCCTTATAGCCTGTCC |
| ANG1 | Forward | AAAATGGGTTTTGGGAATCCCT |
| | Reverse | TCGGCACCGTGTAAGATCAAG |
| ANG2 | Forward | GGACAGTCATCCAACACCGAG |
| | Reverse | GACTCTTCACCAGCGAGGTAG |

| | | |
|---|---|---|
| β-actin | Forward | CATGTACGTTGCTATCCAGGC |
| | Reverse | GCCTTAATGTCACGCACGAT |

Supplementary Table 1. Primer sequence pairs used for RT-PCR analyses.

| Gene | | Source | CAT# | Sequences |
|---|---|---|---|---|
| On-Target plus SMART pool siRNA-human CXCR4 | #1 | Dharmacon Horizon Discovery, Lafayette, USA | J-005139-06 | GAAGCAUGACGGACAAGUA |
| | #2 | Dharmacon Horizon Discovery, Lafayette, USA | J-005139-07 | GGCCUUAUCCUGCCUGGUA |
| | #3 | Dharmacon Horizon Discovery, Lafayette, USA | J-005139-08 | UAACUACACCGAGGAAAUG |
| | #4 | Dharmacon Horizon Discovery, Lafayette, USA | J-005139-09 | CAAGCAAGGGUGUGAGUUU |
| On-Target plus SMART pool siRNA-human CXCR7 | #1 | Dharmacon Horizon Discovery, Lafayette, USA | J-013212-10 | GCCGUUCCCUUCUCCAUUA |
| | #2 | Dharmacon Horizon Discovery, Lafayette, USA | J-013212-11 | UACACGCUCUCCUUCAUUU |
| | #3 | Dharmacon Horizon Discovery, Lafayette, USA | J-013212-12 | GAGCUGGUCUCCGUUGUCU |
| | #4 | Dharmacon Horizon Discovery, Lafayette, USA | J-013212-13 | GCUCAUCGAUGCCUCCAGA |

Supplementary Table 2. Sequences of siRNA.

| Large category | Small category | Term | Term P-value |
|---|---|---|---|
| Biological process (BP) | Embryo implantation | GO:0001835~blastocyst hatching | 2.97E-04 |
| Biological process (BP) | | GO:0007566~embryo implantation | 8.30E-04 |
| Biological process (BP) | Sprouting angiogenesis | GO:0002040~sprouting angiogenesis | 4.85E-08 |
| Biological process (BP) | | GO:0002042~cell migration involved in sprouting angiogenesis | 3.94E-07 |
| Biological process (BP) | | GO:0043534~blood vessel endothelial cell migration | 1.93E-05 |
| Biological process (BP) | Regulation of vasculature development | GO:0016525~negative regulation of angiogenesis | 4.36E-08 |
| Biological process (BP) | | GO:0030947~regulation of vascular endothelial growth factor receptor signaling pathway | 8.87E-05 |
| Biological process (BP) | | GO:0048010~vascular endothelial growth factor receptor signaling pathway | 2.91E-04 |
| Biological process (BP) | | GO:1901342~regulation of vasculature development | 9.17E-16 |
| Biological process (BP) | | GO:1901343~negative regulation of vasculature development | 1.33E-10 |
| Biological process (BP) | | GO:2000181~negative regulation of blood vessel morphogenesis | 5.12E-08 |
| Biological process (BP) | Regulation of epidermal growth factor receptor signaling pathway | GO:0005006~epidermal growth factor-activated receptor activity | 8.87E-05 |
| Biological process (BP) | | GO:0007173~epidermal growth factor receptor signaling pathway | 7.89E-06 |
| Biological process (BP) | | GO:0038127~ERBB signaling pathway | 1.29E-05 |
| Biological process (BP) | | GO:0042058~regulation of epidermal growth factor receptor signaling pathway | 3.65E-05 |
| Biological process (BP) | | GO:0045742~positive regulation of epidermal growth factor receptor signaling pathway | 7.33E-05 |
| Biological process (BP) | | GO:1901184~regulation of ERBB signaling pathway | 4.77E-05 |
| Biological process (BP) | | GO:1901186~positive regulation of ERBB signaling pathway | 8.87E-05 |
| Biological process (BP) | Regulation of collagen metabolic process | GO:0003197~endocardial cushion development | 2.41E-04 |
| Biological process (BP) | | GO:0010712~regulation of collagen metabolic process | 4.70E-07 |
| Biological process (BP) | | GO:0032330~regulation of chondrocyte differentiation | 3.88E-04 |
| Biological process (BP) | | GO:0032331~negative regulation of chondrocyte differentiation | 2.93E-05 |
| Biological process (BP) | | GO:0061037~negative regulation of cartilage development | 7.33E-05 |
| Biological process (BP) | Regulation of muscle contraction | GO:0001990~regulation of systemic arterial blood pressure by hormone | 2.91E-04 |
| Biological process (BP) | | GO:0001991~regulation of systemic arterial blood pressure by circulatory renin-angiotensin | 1.36E-05 |
| Biological process (BP) | | GO:0003014~renal system process | 7.64E-07 |
| Biological process (BP) | | GO:0003044~regulation of systemic arterial blood pressure mediated by a chemical signal | 1.63E-05 |
| Biological process (BP) | | GO:0003071~renal system process involved in regulation of systemic arterial blood pressure | 5.37E-05 |
| Biological process (BP) | | GO:0003073~regulation of systemic arterial blood pressure | 1.11E-06 |
| Biological process (BP) | | GO:0003081~regulation of systemic arterial blood pressure by renin-angiotensin | 1.06E-04 |
| Biological process (BP) | | GO:0003085~negative regulation of systemic arterial blood pressure | 6.64E-05 |
| Biological process (BP) | | GO:0006937~regulation of muscle contraction | 1.14E-08 |
| Biological process (BP) | | GO:0006939~smooth muscle contraction | 5.37E-07 |
| Biological process (BP) | | GO:0006940~regulation of smooth muscle contraction | 2.42E-08 |
| Biological process (BP) | | GO:0035150~regulation of tube size | 3.17E-07 |

| | | | |
|---|---|---|---|
| Biological process (BP) | | GO:0035296~regulation of tube diameter | 3.04E-07 |
| Biological process (BP) | | GO:0042311~vasodilation | 1.25E-04 |
| Biological process (BP) | | GO:0044060~regulation of endocrine process | 1.87E-05 |
| Biological process (BP) | | GO:0045776~negative regulation of blood pressure | 2.57E-05 |
| Biological process (BP) | | GO:0045907~positive regulation of vasoconstriction | 2.57E-04 |
| Biological process (BP) | | GO:0045933~positive regulation of muscle contraction | 1.32E-05 |
| Biological process (BP) | | GO:0045987~positive regulation of smooth muscle contraction | 2.65E-06 |
| Biological process (BP) | | GO:0061045~negative regulation of wound healing | 5.28E-05 |
| Biological process (BP) | | GO:0097746~regulation of blood vessel diameter | 3.04E-07 |
| Biological process (BP) | | GO:0097755~positive regulation of blood vessel diameter | 1.37E-06 |
| Biological process (BP) | | GO:0098801~regulation of renal system process | 4.45E-06 |
| Biological process (BP) | | GO:1900046~regulation of hemostasis | 7.06E-05 |
| Biological process (BP) | | GO:1900047~negative regulation of hemostasis | 3.47E-04 |
| Biological process (BP) | | GO:1903035~negative regulation of response to wounding | 1.19E-04 |
| Biological process (BP) | | GO:1903524~positive regulation of blood circulation | 5.06E-06 |
| Cellular component (CC) | | GO:0005901~caveola | 1.04E-05 |
| KEGG pathway | GnRH secretion | KEGG:04929~GnRH secretion | 6.10E-04 |

**Supplementary Table 3.** Gene Ontology and pathway analysis of a network of gene-gene interaction among CXCL12-regulated genes constructed and visualized in Figure 3F.
