# Supplementary material for: CXCL12 enhances pregnancy outcome via improvement of endometrial receptivity in mice
Source: Sci Rep. 2021 Apr 1;11:7397. doi: 10.1038/s41598-021-86956-y (PMC8016928; doi:10.1038/s41598-021-86956-y)
Supplement: Supplementary file 2 — Supplementary Information 2. [file 41598_2021_86956_MOESM2_ESM.docx]

**Supplementary Figure 1.**

**(A)** A schematic diagram of Ishikawa cells grown on Matrigel-coated coverslip. **(B)** Immunofluorescence (IF) staining images (1-7) were obtained from 7 different regions (illustrated in a diagram of **(B:1-7)**) of coverslip. Red: CXCR4, Blue: DAPI. Intensity of CXCR4 in each image was profiled. Images were captured at a magnification of 10x. **(C)** A schematic diagram of co-culture system of mouse embryo with Ishikawa cells on Matrigel-coated coverslip. **(D)** IF staining of images (1-2) were obtained from 2 different regions (illustrated in a diagram of **(C:1-2)**) of coverslip. Mouse embryo was attached in the region number 1 as illustrated with red spot **(C)** and white arrow **(D)**. Red: CXCR7, Blue: DAPI. Intensity of CXCR7 in each image was profiled. Images were captured at a magnification of 10x.

**Supplementary Figure 2.**

Average numbers of embryo implantation sites with 10ng **(A)** or 50ng **(B)** CXCL12 intra-uterine treatment compared to saline-treated group. Data are from 5 independent female mice, and analyzed using paired t-test analysis including p-values. **(C)** Representative H&E images of the sites of embryo implantation in Control vs. CXCL12-treated mouse uterus. Regions indicated with 1~3 of the image at far-left side were magnified in image #1, #2, and #3. Scale bar; 50um **(D)** A graph showing the number of embryo implantation sites in CXCL12 neutralizing antibody (5ug/horn)-treated uterine horn compared to saline-treated horn. Data are from 4 independent female mice, and analyzed using paired t-test analysis including p-values (P<0.05(*), NS; not significant). QRT-PCR analysis of CXCR4 **(E)** and CXCR7 **(F)** in siControl, siCXCR4, siCXCR7, or siCXCR4/7-transfected Ishikawa cells compared to non-treated cells (Control). Comparison groups were analyzed with the ordinary two-way ANOVA analysis with Dunnett’s multiple comparison test. For all cases, a P-value that was <0.05 was considered statistically significant (P<0.0001(****), NS; not significant). **(G)** Rates of stability of attached mouse embryos in siControl, siCXCR4, siCXCR7, or siCXCR4/7-transfected Ishikawa cells compared to non-treated cells (Control) at 19h, 24h, 28h, or 45h of co-culture. (n of embryos=44)

**Supplementary Table 1.** Primer sequence pairs used for RT-PCR analyses.

**Supplementary Table 2.** Sequences of siRNA.

**Supplementary Table 3.** Gene Ontology and pathway analysis of a network of gene-gene interaction among CXCL12-regulated genes constructed and visualized in Figure 3F.
